# Supplementary material for: Effectiveness of intralesional sodium stibogluconate for the treatment of localized cutaneous leishmaniasis at Boru Meda general hospital, Amhara, Ethiopia: Pragmatic trial
Source: PLoS Negl Trop Dis. 2022 Sep 9;16(9):e0010578. doi: 10.1371/journal.pntd.0010578 (PMC9491591; doi:10.1371/journal.pntd.0010578)
Supplement: S1 CONSORT Checklist — (DOCX) [file pntd.0010578.s001.docx]

**Box A in S1: CONSORT Statement—Checklist of items for reporting pragmatic trials**

| Section | Item | Standard CONSORT description | Paragraphs in the Manuscript |
| --- | --- | --- | --- |
| Title and abstract | 1 | How participants were allocated to interventions (eg, “random allocation,” “randomised,” or “randomly assigned”) | Abstract: methodology/principal findings |
| **Introduction** |  |  |  |
| Background | 2 | Scientific background and explanation of rationale | Paragraph 1-5 |
| **Methods** |  |  |  |
| Participants | 3 | Eligibility criteria for participants; settings and locations where the data were collected | *Method section:*Sub-section: study design, population, and recruitment |
| Interventions | 4 | Precise details of the interventions intended for each group and how and when they were actually administered | *Method section:*  *Subsection:* Routine diagnosis, treatment and follow-up (Paragraph 1 and 2) and study procedures (paragraph 1) |
| Objectives | 5 | Specific objectives and hypotheses | Introduction: paragraph 5 |
| Outcomes | 6 | Clearly defined primary and secondary outcome measures and, when applicable, any methods used to enhance the quality of measurements (eg, multiple observations, training of assessors) | *Method section:*  *Sub-section:* Routine diagnosis, treatment and follow-up (Paragraph 2), study procedures (paragraph 1), and data collection and analysis (paragraph 1) |
| Sample size | 7 | How sample size was determined; explanation of any interim analyses and stopping rules when applicable | *Method section:*  *Sub-section:* Study design, population, and recruitment (paragraph 1) |
| Randomisation—sequence generation | 8 | Method used to generate the random allocation sequence, including details of any restriction (eg, blocking, stratification) | *Not Applicable* |
| Randomisation—allocation concealment | 9 | Method used to implement the random allocation sequence (eg, numbered containers or central telephone), clarifying whether the sequence was concealed until interventions were assigned | *Not applicable* |
| Randomisation—implementation | 10 | Who generated the allocation sequence, who enrolled participants, and who assigned participants to their groups | *Not applicable* |
| Blinding (masking) | 11 | Whether participants, those administering the interventions, and those assessing the outcomes were blinded to group assignment | Not applicable |
| Statistical methods | 12 | Statistical methods used to compare groups for primary outcomes; methods for additional analyses, such as subgroup analyses and adjusted analyses | *Not applicable* |
| **Results** |  |  |  |
| Participant flow | 13 | Flow of participants through each stage (a diagram is strongly recommended)—specifically, for each group, report the numbers of participants randomly assigned, receiving intended treatment, completing the study protocol, and analysed for the primary outcome; describe deviations from planned study protocol, together with reasons | *Result section:*  *Sub-section:* Treatment and outcome (figure 1) |
| Recruitment | 14 | Dates defining the periods of recruitment and follow-up | *Method section:*  *Sub-section:* Routine diagnosis, treatment and follow-up (Paragraph 2), and  *Result section:*  *Sub-section:* Treatment and outcome (figure 1), Table-4 |
| Baseline data | 15 | Baseline demographic and clinical characteristics of each group | *Result section:* *Sub-section:* Patient characteristics (paragraph 1-5 and Table-1) |
| Numbers analysed | 16 | Number of participants (denominator) in each group included in each analysis and whether analysis was by “intention-to-treat”; state the results in absolute numbers when feasible (eg, 10/20, not 50%) | *Result section:*  *Sub-section:* Patient characteristics and Treatment and outcome  *Methods section:*  *Sub-section:* Data analysis: PP analysis |
| Outcomes and estimation | 17 | For each primary and secondary outcome, a summary of results for each group and the estimated effect size and its precision (eg, 95% CI) | *Result section:*  *Sub-section:* Patient characteristics and Treatment and outcome (Paragraph 4, and Table 3) |
| Ancillary analyses | 18 | Address multiplicity by reporting any other analyses performed, including subgroup analyses and adjusted analyses, indicating which are pre-specified and which are exploratory | *Not applicable* |
| Adverse events | 19 | All important adverse events or side effects in each intervention group | *Result section:*  *Sub-section:* Treatment and outcome (Paragraph 1) |
| **Discussion** |  |  |  |
| Interpretation | 20 | Interpretation of the results, taking into account study hypotheses, sources of potential bias or imprecision, and the dangers associated with multiplicity of analyses and outcomes | *Discussion section*  (Paragraph 1-6) |
| Generalisability | 21 | Generalisability (external validity) of the trial findings | *Discussion section*  (Paragraph 2-5) |
| Overall evidence | 22 | General interpretation of the results in the context of current evidence | *Discussion section*  (Paragraph 1-4) |

***Cite as:*** *Zwarenstein M, Treweek S, Gagnier JJ, Altman DG, Tunis S, Haynes B, Oxman AD, Moher D for the CONSORT and Pragmatic Trials in Healthcare (Practihc) group. Improving the reporting of pragmatic trials: an extension of the CONSORT statement. BMJ 2008; 337;a2390.*
