# Supplementary material for: Effectiveness of intralesional sodium stibogluconate for the treatment of localized cutaneous leishmaniasis at Boru Meda general hospital, Amhara, Ethiopia: Pragmatic trial
Source: PLoS Negl Trop Dis. 2022 Sep 9;16(9):e0010578. doi: 10.1371/journal.pntd.0010578 (PMC9491591; doi:10.1371/journal.pntd.0010578)
Supplement: S1 Tables. Table A — Table B: Lesion summary tables of missed follow-up LCL patients, Boru Meda general hospital, 2021. Table C: Missed follow-up patients’ description and clinical profiles for IL SSG treating LCL, Boru Meda general hospital, 2021. Table D: The fit and residual values of the models of effectiveness of IL SSG treating LCL, Boru meda general hospital, 2021. (DOCX) [file pntd.0010578.s002.docx]

# Table A in S1 Tables. Measurements of LCL Before and while ongoing treatment (follow ups), Boru meda general Hospital, 2021.

| Measurements | | Before | After |
| --- | --- | --- | --- |
| Size of the lesion (cm) | Mean ± SD | 3.165 ± 0.96 | 2.00 ± 1.14 |
|  | Minimum | 1.0 | 0.00 |
|  | Maximum | 5.0 | 4.5 |

Missed follow up patients descriptions and clinical profiles

We have a total of 11 patients who missed their IL SSG treatment follow-up for LCL more than 2 doses. Their median age was 2.25 ±1.5 (mean ± SD) (Table A in S1 Tables). Among missed patients 54.5% of the patients were male, 90.9% were age less than 45years, 54.5% were students at the elementary level (45.5%), and 8 (72.7%) lesion sites were at the extremities. All patients who missed the follow-up had a treatment history from the health institution but more than half (7(63.6%)) of the patients did not use traditional treatment (See table 8). But all patients who missed their follow-up the size of the lesion were less than 2cm at the index lesions.

Table B in S1 Tables. Lesion summary tables of missed follow-up LCL patients, Boru Meda general hospital, 2021.

|  | Median |
| --- | --- |
| Age | 2.25 ± 1.5 |
| Size of lesion | 1.71 ± 0.46 |
| Number of lesion | 1.09 ± 0.3 |

Table C in S1 Tables**.** Missed follow-up patients’ description and clinical profiles for IL SSG treating LCL, Boru Meda general hospital, 2021.

| Characteristics | | N (%) |
| --- | --- | --- |
| Sex | Male | 6 (54.5%) |
|  | Female | 5 (45.5%) |
| Age | Less than 45 | 10(90.9) |
|  | Above 45 | 1 (9.1%) |
| Residence | Rural | 4 (36.4%) |
|  | Urban | 7(63.6%) |
| Occupation | Student | 6 (54.5%) |
|  | Farmer | 2 (18.2%) |
|  | Civil servant | 2 (18.2%) |
|  | Merchant | 1 (9.1%) |
| Educational status | Elementary | 5 (45.5%) |
|  | High school and preparatory | 3(27.3%) |
|  | Diploma and above | 2 (18.2%) |
|  | Illiterate | 1(9.1%) |
| Traditional medicine | Yes | 4 (36.4%) |
|  | No | 7(63.6%) |
| Duration of the lesion | </=1year | 8 (72.8%) |
|  | >1year | 3 (27.3%) |
| No of the lesion | Single | 10 (90.9%) |
|  | Two | 1 (9.1%) |

**Modeling the final outcome (Multi-level logistic Regressions)**

To begin with, the first model to be tested as an entry to use a multi-level model is the null model (unconditional mean). From the null model, Intra-class Correlation Coefficient (ICC) which is the variability among intercepts relative to total variability is calculated to be 99.9% for the null model. This value is greater than the minimum recommended value to use a multilevel model. The high value of ICC had also further strengthened the appropriateness of using a multilevel model for the data. Therefore, the modeling process was started with the null model.

Next to the null model, the logistic model containing a random intercept and measurements to be constant as both fixed and random effect variable was fitted. In this second model, the fixed intercept were 1.58% were statistically significant with a p value of 0.005. The third model included the combined predictors from different levels (model one and model 2) to explain the variability among effectiveness of the treatment (IL SSG).

Table D in S1 Tables. The fit and residual values of the models of effectiveness of IL SSG treating LCL, Boru meda general hospital, 2021

| Model | BIC | AIC | df | Residual |
| --- | --- | --- | --- | --- |
| Null model (Model One) | 110.077 | 82.757 | 2 | 82.39 |
| Model Two | 113.193 | 90.426 | 10 | 3.18 |
| Model Three (combined) | 97.739 | 93.186 | 12 | 1.58 |

After computing the models, selection of the models to be used and the result to be interpreted was selected based on the AIC and BIC Accordingly, when the null model was compared with the second model, there was a statistically significant difference because the change in AIC and BIC and this was significant at Chi square with 10 degrees of freedom. The residual error variance also dropped from 82.39 to 3.18, implying that about 80.81% of the within individual variation in the effectiveness was associated with the effect of independent variables considered. Therefore, taking the smallest value of AIC and BIC from the model containing the predictors, we took model three as best fitted model (Table D in S1 Tables).
