# Supplementary material for: Effectiveness of intralesional sodium stibogluconate for the treatment of localized cutaneous leishmaniasis at Boru Meda general hospital, Amhara, Ethiopia: Pragmatic trial
Source: PLoS Negl Trop Dis. 2022 Sep 9;16(9):e0010578. doi: 10.1371/journal.pntd.0010578 (PMC9491591; doi:10.1371/journal.pntd.0010578)
Supplement: Attachment — Submitted filename: 3-Response to Reviewers.docx [file pntd.0010578.s004.docx]

The manuscript “Effectiveness of intralesional sodium stibogluconate (IL SSG) for the treatment of localized cutaneous leishmaniasis at Boru Meda general hospital, Amhara, Ethiopia:

Pragmatic trial” by Zewdu et al is an article assessing the treatment effectiveness of intralesional sodium stibogluconate for cutaneous leishmaniasis. This is an important area of research for countries where cutaneous leishmaniasis is endemic and treatments are not widely available or effective. The article would benefit from extensive editing and additional statistical model assessment review.

**Major Comments:**

Overall

1. Please note the abstract entered into the abstract category box is not the same as the abstract written in the paper. Some of the odds ratios and numbers were not transcribed correctly.

- Thank you for noticing this. Unfortunately, the abstract in the category box (and the title) were not updated. The correct versions were in the main manuscript. This has been corrected now.

Introduction

1. The researchers state there is no data from Ethiopia on sodium stibogluconate effectiveness but this is incorrect. The first author alone published data in 2018 looking at the treatment effectiveness of intralesional SSG in comparison to other treatments for different stages of leishmaniasis. Furthermore, another additional article from 2012 looked at intralesional SSG vs cryotherapy. The authors should edit the abstract and introduction to better attribute that while there is data available it is limited and additional data is necessary to improving our understanding of intralesional sodium stibogluconate treatment effectiveness.

- Accepted and corrected accordingly
- The references also added to the area where needed to be cited.

2. Line 81: The authors as well as others have published smaller studies on the usage of intralesional SSG these should be referenced here as well. Examples are listed below:
   a. Treatment response of cutaneous leishmaniasis due to Leishmania aethiopica to cryotherapy and generic sodium stibogluconate from patients in Silti, Ethiopia by Negera et al. Transactions of the Royal Society of Tropical Medicine & Hygiene. 2012
   b. Efficacy and safety of a combined treatment of sodium stibogluconate at 20mg/kg/day with upper maximum daily dose limit of 850mg and Paromomycin 15mg/kg/day in HIV negative visceral leishmaniasis patients. A retrospective study, northwest Ethiopia. Tamiru et al. 2021 PLOS Neglected Tropical Diseases.
   c. Zewdu T, Seife T, Ayal A, Misganaw M (2018) Treatment Patterns, And Effectivness of Anti-Leishmaniasis Agents for Page 6 of 6 Patients with Cutaneous Leishmaniasis at Boru Meda Hospital, South Wollo, North East Ethiopia, 2017/18. Clin Res Dermatol Open Access 5(2): 1-6. DOI: <http://dx.doi.org/10.15226/2378-1726/5/2/00179>

- Thank you for these suggestions. Reference a) and b) concern systemic use of sodium stibogluconate, and are therefore not directly relevant. We have chosen not to use them here. We have added a few lines where we describe the evidence generated in c), and why this study was not sufficient to show effectiveness of IL SSG in Ethiopian patients

3. Line 82. The authors reference anecdotal reports but no evidence or further discussion. Several studies have reported adverse events when treating with sodium stibogluconate so these could be discussed in this section of the introduction.

- Thank you for this comment. An important distinction to be made is between systemic SSG treatment and intralesional SSG treatment. For systemic SSG side-effects have indeed been reported in many different studies, but evidence on intralesional SSG (especially in Ethiopia) is much less clear. We have changed the phrasing of the introduction to reflect this more clearly.

Methods

1. Skin slit smear method needs to be described and references included.

- The references also added

2. Statistical methods should not be supplemental documents. They should be included in the main manuscript.

- removed from the supplemental documents and included to the main body of the manuscript

3. Regarding model selection the authors chose to use BIC criteria but also referenced AIC the authors need to first include the written term Akaike Information Criterion but also need to provide reasoning for using the BIC instead of the AIC in determining model fit. Furthermore based on the tables the authors fitted just demographic factors in the 2^nd^ model and then removed occupational factors but added the skin slit test results for model three. This is therefore not a true combination of models one and two as is suggested in the text. The authors need to be clear about this and provide further description for how variables were chosen to be included or removed from the model. Since model three was not a true addition of the first two models why other models were where other variables were removed not tested. Furthermore, why was a stepwise method, for example, not employed to identify the most appropriate final model with lowest combined BIC and AIC.

- First, we used both of the model selection (Table 4 Supplemental documents). Unfortunately we wrote the BIC selection criteria only.
- Furthermore, theoretically we know tables should not be more than one page. So in order to keep this we added and removed the variables whenever no associated with the outcome variables. No more reason to add and remove such variables in the tables, please.
- In conclusion, the variable will be included in the model depending on the nature of the variables. i.e repeated measured fixed (repeated) variables (Further, see Supplemental documents).

**Minor Edits:**

Overall

1. There are several grammatical errors throughout the paper. The paper would benefit from additional review for grammatical errors and flow.

- We apologize for this. We have tried to improve on this by carefully reviewing the manuscript again with help from the co-authors. We hope this is deemed sufficient.
- We have tried to edit and corrected using a scrupulous reading and grammar and spelling checker software (Grammarly Add In Set up).

Abstract

1. Line 32: SSS acronym listed with no description.

- Done

2. The authors state in the abstract cryotherapy vs sodium stibogluconate has not been assessed in Ethiopia. Further research is necessary but the authors need to appropriate reference in the abstract that research has been completed surrounding that topic. The authors even mention in the discussion section some of the preliminary studies surrounding both of these treatments.

- This has been clarified in the abstract.

Introduction

- Line 73: Reference 25: This reference does not list what agency the guidelines are from nor does it include reference link or other identifier for readers to view the reference document.
  - The guidelines that we referred to are the national leishmaniasis treatment guidelines. We have clarified this in line 80.

Methods

1. Line 95: “In conclusion” is not the appropriate wording for this sentence.

- Edited and corrected

2. Line 108: Please list the specific guideline documents referenced in this sentence.

- We have added these to the reference list

3. Line 114: Please list what the abroad-based research recommendations are specifically and provide appropriate references.

- We have added and discussed this

4. Line 133: The authors state that they utilized CTCAE to identify adverse event with some modification. The authors should list what modifications were used. Furthermore, modification is currently spelled wrong. Mod**e**fications should be mod**i**fications.

- We have simplified the CTCAE classification by mild (grade I), moderate (grade II) and severe (grade III).
- Spelling error= Corrected, thank you for pointing this out.
- Line 139. Please reference the products used properly. For example SPSS: IBM Corp. Released 2011. IBM SPSS Statistics for Windows, Version 20.0. Armonk, NY: IBM Corp. STATS: StataCorp. 2015. Stata Statistical Software: Release 14. College Station, TX: StataCorp LP. Please note, most statistical software companies provide examples for appropriate citation of their products in research publications.
- Added/Done
- Line 142 and 144: There are several grammatical errors within these sentences. Please address. For example: This could read “Adherence was determined by whether the patient received ≥ 4 doses.” Currently this sentence is contradictory listing adherence as “patient who had a follow up ≥ 4 doses or less.”
- Tried to correct and edited to make paragraph to be easily understandable.
- Please provide evidence supporting why cure status (cure, good treatment response, partial treatment response, no treatment response) were classified into the categories listed. Discussion of how these categories compare to other studies assessing sodium stibogluconate treatment effectiveness should be included in the manuscript.
- We have added a couple of our references used

Results

1. Line 181: At the end of this sentence the wording should read “the farthest came from 140 km away.”

- Removed due to its less significance

2. Line 190: Please re-word to read “used treatments from the referral health institution.”

- Corrected

3. Line 207: This sentence needs to be rewritten for clarity.

- Corrected

4. Line 209: This sentence needs to be rewritten there are spacing and parenthesis errors.

- Corrected

5. Line 221: This sentence needs to be rewritten for clarity.
6. Line 223: Please add an e to the word respons. Should be “response.”

- Corrected

7. Line 226: Please re-word the sentence to read “or were provided a follow-up appointment for re-evaluation.”

- Corrected

Discussion

1. Line 283: This sentence needs to be rewritten for clarity.
2. Lines 309-314: Please provide additional sentences to improve the clarity of how this paragraph fits into the discussion and how it affects your results.

- We have tried to correct and edited the paragraph.

**Some of the changes made were:**

Added sample size calculation

Added consort guideline and adapted accordingly

Clarified outcome definitions

Updated references

Added paragraph in discussion on skin slit results
